# 7-[(Morpholin-4-yl)(phen­yl)meth­yl]quinolin-8-ol

**DOI:** 10.1107/S1600536812049343

**Published:** 2012-12-08

**Authors:** J. Josephine Novina, G. Vasuki, C. Muthukumar, A. Sabastiyan, A. Crochet, K. M. Fromm

**Affiliations:** a Department of Physics, Idhaya College for Women, Kumbakonam-1, India; b Department of Physics, Kunthavai Naachiar Govt. Arts College (W) (Autonomous), Thanjavur-7, India; c Department of Chemistry, Urumu Dhanalakshmi College, Tiruchirappalli-19, India; d Fribourg Center for Nanomaterials, FriMat, University of Fribourg, Switzerland; e Department of Chemistry, University of Fribourg, Switzerland

## Abstract

In the title compound, C_20_H_20_N_2_O_2_, the quinoline ring system makes dihedral angles of 81.05 (4) and 61.16 (5)° with the mean planes of the benzene and morpholine rings, respectively; the mean planes of the latter two rings make a dihedral angle of 83.59 (4)°. In the crystal, pairs of O—H⋯N hydrogen bonds link neighbouring mol­ecules related by a twofold rotation axis, generating *R*_2_^2^(10) motifs.

## Related literature

For the biological activity of quinoline derivatives, see: Thakur *et al.* (2010 ▶). For hydrogen-bond motifs, see: Bernstein *et al.* (1995 ▶). For puckering parameters, see: Cremer & Pople (1975 ▶).
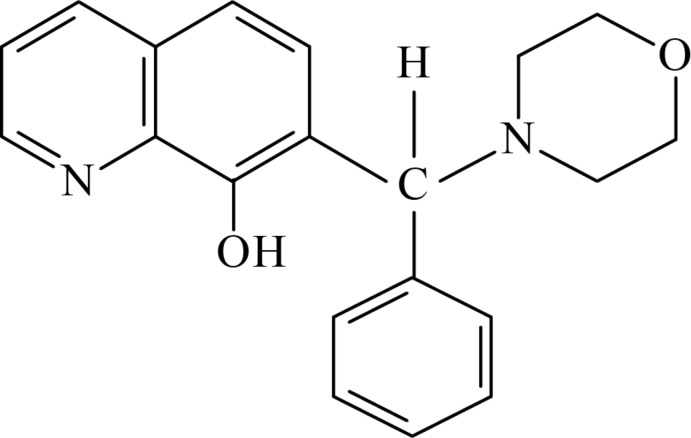


## Experimental

### 

#### Crystal data

C_20_H_20_N_2_O_2_
*M* *_r_* = 320.38
Orthorhombic, 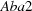

*a* = 13.1537 (6) Å
*b* = 31.0875 (13) Å
*c* = 8.3175 (3) Å
*V* = 3401.2 (2) Å^3^
*Z* = 8
Cu *K*α radiation
μ = 0.65 mm^−1^
*T* = 200 K
0.60 × 0.32 × 0.17 mm

#### Data collection

Stoe IPDS 2 diffractometer
Absorption correction: integration (*X-SHAPE*: Stoe & Cie, 2002 ▶) *T*_min_ = 0.696, *T*_max_ = 0.898
12678 measured reflections
2776 independent reflections
2696 reflections with *I* > 2σ(*I*)
*R*_int_ = 0.074

#### Refinement

*R*[*F* ^2^ > 2σ(*F* ^2^)] = 0.028
*wR*(*F* ^2^) = 0.074
*S* = 1.06
2776 reflections
218 parameters
1 restraint
H atoms treated by a mixture of independent and constrained refinement
Δρ_max_ = 0.16 e Å^−3^
Δρ_min_ = −0.14 e Å^−3^
Absolute structure: Flack (1983 ▶), 1273 Friedel pairs
Flack parameter: −0.05 (18)

### 

Data collection: *X-AREA* (Stoe & Cie, 2002 ▶); cell refinement: *X-AREA*; data reduction: *X-RED32* (Stoe & Cie, 2002 ▶); program(s) used to solve structure: *SHELXS97* (Sheldrick, 2008 ▶); program(s) used to refine structure: *SHELXL97* (Sheldrick, 2008 ▶); molecular graphics: *ORTEP-3* (Farrugia, 2012 ▶) and *Mercury* (Macrae *et al.*, 2008 ▶); software used to prepare material for publication: *PLATON* (Spek, 2009 ▶).

## Supplementary Material

Crystal structure: contains datablock(s) I, global. DOI: 10.1107/S1600536812049343/gk2537sup1.cif

Structure factors: contains datablock(s) I. DOI: 10.1107/S1600536812049343/gk2537Isup2.hkl

Supplementary material file. DOI: 10.1107/S1600536812049343/gk2537Isup3.cml

Additional supplementary materials:  crystallographic information; 3D view; checkCIF report

## Figures and Tables

**Table 1:** Hydrogen-bond geometry (Å, °)

*D*—H⋯*A*	*D*—H	H⋯*A*	*D*⋯*A*	*D*—H⋯*A*
O1—H1*A*⋯N1^i^	0.85 (2)	2.01 (2)	2.7668 (14)	148 (18)

Symmetry code: (i) 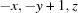.

**Fig. 1.**
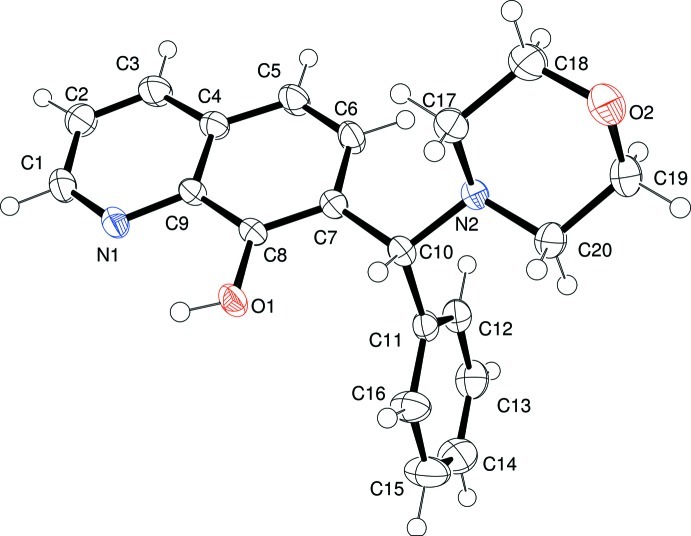
The molecular structure of the title compound with displacement ellipsoids drawn at the 50% probability level.

**Fig. 2.**
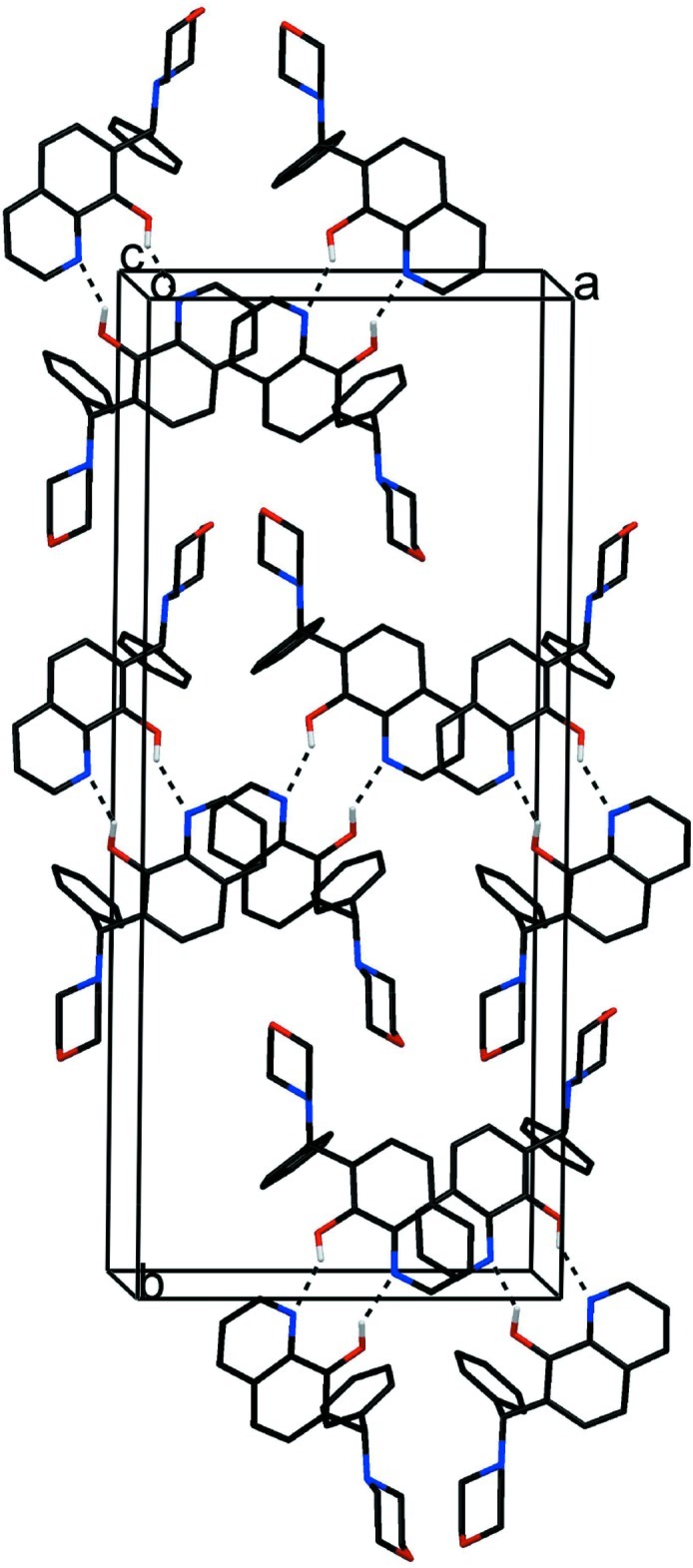
Crystal packing of the title compound viewed down the c axis. Dashed lines indicate hydrogen bonds. H atoms not involved in hydrogen bonding have been omitted.

## supplementary crystallographic information

### Comment

Quinoline analogues have been reported to display promising biological activities such as antimicrobial, anti–inflammatoty, antileishmanial, antituberculosis, antimalarial, cytotoxicity and HIV–1 integrase inhibitors (Thakur *et al.*, 2010). In continuation of our efforts to develop quinoline derivatives with a new structure–activity relationalship, herein, we report the synthesis and structure determination of the title compound.

In the title molecule (Fig.1), the benzene(C11—C16) and morpholine (N2/O2/C17—C20) rings make dihedral angles of 81.05 (4)° and 61.16 (5)° with the quinoline ring system, respectively. The dihedral angle between the benzene and morpholine rings is 83.59 (4)°. The title molecule is chiral with a chiral centre at C10. The morpholine ring adopts an almost perfect normal chair conformation having total puckering amplitude, Q_T_ of 0.5876 (15) Å, θ = 3.34 (14)° and φ = 176 (3)° (Cremer & Pople, 1975). The sum of the bond angles around N2 [329.13 (32)°] indicates a pyramidal geometry. The N2 atom deviates by 0.2613 (10) Å from the least–squares plane passing through atoms C17—C20.

In the crystal packing (Fig. 2), intermolecular O—H···N hydrogen bonds (Table 1) link the neighbouring molecules and generate an *R*_2_^2^(10) motif (Bernstein *et al.*, 1995).

At x=0.0, y= 0.0, z= 0.321 the crystal contains small void with the solvent accessible volume of 33 Å^3^.

### Experimental

8-Hydroxyquinoline (14.5 g; 0.1 mol) was dissolved in 25 ml of acetone and placed in a 250 ml beaker with constant stirring at 305 K for 1/2 h, then benzaldehyde (10.6 g; 0.1 mol) followed by morpholine (8.71 g; 0.1 mol) was added and the mixture was stirred well. The reaction mixture was left for 48 h and the resulting precipitate was collected by filtration and washed with cold water to give pale brown solid (m.p. 338 K) was obtained (yield: 70%). Single crystals suitable for X-ray diffraction were obtained from ethanol.

### Refinement

H1A of the OH group was located in an electron difference map and refined freely. Remaining H atoms were positioned geomentrically and allowed to ride on their parent atoms, with C—H = 0.95–1.00 Å and *U*_iso_(H) = 1.2 times *U*_eq_(C).

### Crystal data

**Table:**

C_20_H_20_N_2_O_2_	*F*(000) = 1360
*M**_r_* = 320.38	*D*_x_ = 1.251 Mg m^−^^3^
Orthorhombic, *A**b**a*2	Cu *K*α radiation, λ = 1.54186 Å
Hall symbol: A 2 -2ac	Cell parameters from 29594 reflections
*a* = 13.1537 (6) Å	θ = 3.4–67.3°
*b* = 31.0875 (13) Å	µ = 0.65 mm^−^^1^
*c* = 8.3175 (3) Å	*T* = 200 K
*V* = 3401.2 (2) Å^3^	Block, brown
*Z* = 8	0.60 × 0.32 × 0.17 mm

### Data collection

**Table:**

Stoe IPDS 2 diffractometer	2776 independent reflections
Radiation source: fine-focus sealed tube	2696 reflections with *I* > 2σ(*I*)
Graphite monochromator	*R*_int_ = 0.074
Detector resolution: 6.67 pixels mm^-1^	θ_max_ = 64.9°, θ_min_ = 4.4°
rotation method scans	*h* = −15→15
Absorption correction: integration (*X-SHAPE*: Stoe & Cie, 2002)	*k* = −34→34
*T*_min_ = 0.696, *T*_max_ = 0.898	*l* = −9→9
12678 measured reflections	

### Refinement

**Table:**

Refinement on *F*^2^	Secondary atom site location: difference Fourier map
Least-squares matrix: full	Hydrogen site location: inferred from neighbouring sites
*R*[*F*^2^ > 2σ(*F*^2^)] = 0.028	H atoms treated by a mixture of independent and constrained refinement
*wR*(*F*^2^) = 0.074	*w* = 1/[σ^2^(*F*_o_^2^) + (0.0315*P*)^2^ + 1.0277*P*] where *P* = (*F*_o_^2^ + 2*F*_c_^2^)/3
*S* = 1.06	(Δ/σ)_max_ < 0.001
2776 reflections	Δρ_max_ = 0.16 e Å^−^^3^
218 parameters	Δρ_min_ = −0.14 e Å^−^^3^
1 restraint	Absolute structure: Flack (1983), 1273 Friedel pairs
Primary atom site location: structure-invariant direct methods	Flack parameter: −0.05 (18)

### Special details

**Table:**

Geometry. All esds (except the esd in the dihedral angle between two l.s. planes) are estimated using the full covariance matrix. The cell esds are taken into account individually in the estimation of esds in distances, angles and torsion angles; correlations between esds in cell parameters are only used when they are defined by crystal symmetry. An approximate (isotropic) treatment of cell esds is used for estimating esds involving l.s. planes.
Refinement. Refinement of F^2^ against ALL reflections. The weighted R-factor wR and goodness of fit S are based on F^2^, conventional R-factors R are based on F, with F set to zero for negative F^2^. The threshold expression of F^2^ > 2sigma(F^2^) is used only for calculating R-factors(gt) etc. and is not relevant to the choice of reflections for refinement. R-factors based on F^2^ are statistically about twice as large as those based on F, and R- factors based on ALL data will be even larger.

### Fractional atomic coordinates and isotropic or equivalent isotropic displacement parameters (Å^2^)

**Table:**

	*x*	*y*	*z*	*U*_iso_*/*U*_eq_	
H1A	0.0489 (15)	0.4676 (7)	0.270 (3)	0.055 (6)*	
O1	0.05418 (7)	0.44234 (3)	0.30675 (14)	0.0289 (2)	
O2	0.17007 (9)	0.23324 (3)	0.25602 (15)	0.0419 (3)	
N1	−0.11876 (8)	0.47965 (3)	0.18343 (15)	0.0273 (3)	
N2	0.07402 (8)	0.30989 (3)	0.37537 (14)	0.0238 (2)	
C1	−0.20163 (10)	0.49659 (4)	0.1212 (2)	0.0329 (3)	
H1	−0.2005	0.5263	0.0942	0.040*	
C2	−0.29128 (11)	0.47365 (5)	0.0923 (2)	0.0371 (4)	
H2	−0.3488	0.4875	0.0464	0.045*	
C3	−0.29459 (11)	0.43097 (5)	0.1313 (2)	0.0357 (3)	
H3	−0.3549	0.4148	0.1137	0.043*	
C4	−0.20791 (10)	0.41094 (4)	0.19806 (18)	0.0272 (3)	
C5	−0.20411 (10)	0.36710 (4)	0.24473 (19)	0.0319 (3)	
H5	−0.2622	0.3493	0.2311	0.038*	
C6	−0.11734 (10)	0.35043 (4)	0.30904 (18)	0.0285 (3)	
H6	−0.1166	0.3211	0.3412	0.034*	
C7	−0.02833 (10)	0.37537 (4)	0.32952 (16)	0.0225 (3)	
C8	−0.03074 (9)	0.41828 (4)	0.28592 (16)	0.0214 (3)	
C9	−0.12057 (10)	0.43685 (4)	0.22082 (16)	0.0230 (3)	
C10	0.06862 (9)	0.35690 (4)	0.40229 (17)	0.0224 (3)	
H10	0.1280	0.3705	0.3472	0.027*	
C11	0.07299 (9)	0.36868 (4)	0.57945 (16)	0.0234 (3)	
C12	−0.00026 (10)	0.35446 (4)	0.68860 (19)	0.0284 (3)	
H12	−0.0524	0.3355	0.6533	0.034*	
C13	0.00197 (11)	0.36756 (5)	0.8477 (2)	0.0364 (3)	
H13	−0.0482	0.3575	0.9206	0.044*	
C14	0.07713 (9)	0.39534 (4)	0.90078 (15)	0.0433 (4)	
H14	0.0784	0.4046	1.0096	0.052*	
C15	0.15029 (9)	0.40941 (4)	0.79385 (15)	0.0460 (4)	
H15	0.2025	0.4282	0.8296	0.055*	
C16	0.14802 (12)	0.39625 (5)	0.6350 (2)	0.0356 (4)	
H16	0.1987	0.4063	0.5628	0.043*	
C17	0.08639 (12)	0.30128 (5)	0.20274 (19)	0.0326 (3)	
H17A	0.1505	0.3143	0.1640	0.039*	
H17B	0.0294	0.3143	0.1423	0.039*	
C18	0.08844 (13)	0.25335 (5)	0.1737 (2)	0.0419 (4)	
H18A	0.0234	0.2406	0.2103	0.050*	
H18B	0.0951	0.2478	0.0570	0.050*	
C19	0.16175 (12)	0.24197 (5)	0.4233 (2)	0.0374 (4)	
H19A	0.2197	0.2286	0.4803	0.045*	
H19B	0.0984	0.2289	0.4650	0.045*	
C20	0.16062 (10)	0.28999 (5)	0.45816 (19)	0.0305 (3)	
H20A	0.1549	0.2949	0.5754	0.037*	
H20B	0.2248	0.3032	0.4205	0.037*	

### Atomic displacement parameters (Å^2^)

**Table:**

	*U*^11^	*U*^22^	*U*^33^	*U*^12^	*U*^13^	*U*^23^
O1	0.0244 (4)	0.0184 (5)	0.0438 (6)	−0.0052 (3)	−0.0056 (4)	0.0051 (4)
O2	0.0467 (6)	0.0311 (5)	0.0478 (7)	0.0138 (5)	−0.0028 (5)	−0.0093 (5)
N1	0.0302 (6)	0.0186 (5)	0.0330 (6)	0.0001 (4)	−0.0026 (5)	0.0014 (5)
N2	0.0266 (5)	0.0184 (5)	0.0264 (6)	0.0027 (4)	−0.0018 (4)	−0.0012 (4)
C1	0.0367 (7)	0.0213 (7)	0.0408 (8)	0.0016 (6)	−0.0069 (7)	0.0043 (6)
C2	0.0339 (7)	0.0315 (8)	0.0461 (10)	0.0042 (6)	−0.0145 (7)	0.0012 (7)
C3	0.0294 (7)	0.0309 (7)	0.0467 (9)	−0.0037 (6)	−0.0110 (6)	−0.0011 (7)
C4	0.0261 (6)	0.0235 (6)	0.0318 (7)	−0.0024 (5)	−0.0038 (6)	−0.0012 (6)
C5	0.0251 (6)	0.0236 (7)	0.0471 (10)	−0.0068 (5)	−0.0065 (6)	0.0010 (6)
C6	0.0288 (7)	0.0189 (6)	0.0377 (8)	−0.0030 (5)	−0.0013 (6)	0.0038 (6)
C7	0.0246 (6)	0.0198 (6)	0.0230 (6)	−0.0003 (5)	0.0012 (5)	−0.0006 (5)
C8	0.0230 (6)	0.0178 (6)	0.0235 (6)	−0.0032 (5)	0.0012 (5)	−0.0014 (5)
C9	0.0265 (6)	0.0178 (6)	0.0246 (7)	−0.0013 (5)	0.0000 (5)	−0.0022 (5)
C10	0.0224 (6)	0.0180 (6)	0.0269 (7)	−0.0010 (5)	0.0019 (5)	0.0000 (5)
C11	0.0248 (6)	0.0188 (6)	0.0265 (7)	0.0028 (5)	−0.0005 (5)	0.0016 (5)
C12	0.0264 (6)	0.0256 (7)	0.0333 (7)	0.0013 (5)	0.0014 (6)	0.0029 (6)
C13	0.0398 (8)	0.0382 (8)	0.0313 (8)	0.0021 (6)	0.0080 (7)	0.0062 (7)
C14	0.0573 (10)	0.0468 (9)	0.0258 (8)	−0.0023 (8)	0.0003 (7)	−0.0043 (7)
C15	0.0552 (9)	0.0495 (10)	0.0331 (9)	−0.0194 (8)	−0.0039 (8)	−0.0075 (8)
C16	0.0390 (8)	0.0402 (8)	0.0276 (8)	−0.0141 (6)	0.0005 (6)	−0.0010 (7)
C17	0.0399 (7)	0.0281 (7)	0.0298 (8)	0.0045 (6)	−0.0026 (6)	−0.0033 (6)
C18	0.0494 (9)	0.0336 (8)	0.0428 (9)	0.0096 (7)	−0.0097 (8)	−0.0104 (7)
C19	0.0392 (8)	0.0282 (7)	0.0449 (9)	0.0111 (6)	−0.0039 (7)	0.0006 (7)
C20	0.0278 (7)	0.0290 (7)	0.0346 (8)	0.0066 (6)	−0.0040 (6)	−0.0004 (6)

### Geometric parameters (Å, º)

**Table:**

O1—C8	1.3554 (15)	C10—C11	1.5195 (18)
O1—H1A	0.85 (2)	C10—H10	1.0000
O2—C18	1.4185 (19)	C11—C16	1.386 (2)
O2—C19	1.422 (2)	C11—C12	1.3957 (19)
N1—C1	1.3165 (18)	C12—C13	1.385 (2)
N1—C9	1.3668 (17)	C12—H12	0.9500
N2—C20	1.4680 (17)	C13—C14	1.385 (2)
N2—C17	1.4696 (19)	C13—H13	0.9500
N2—C10	1.4802 (16)	C14—C15	1.3815
C1—C2	1.399 (2)	C14—H14	0.9500
C1—H1	0.9500	C15—C16	1.383 (2)
C2—C3	1.367 (2)	C15—H15	0.9500
C2—H2	0.9500	C16—H16	0.9500
C3—C4	1.4125 (19)	C17—C18	1.510 (2)
C3—H3	0.9500	C17—H17A	0.9900
C4—C9	1.4157 (18)	C17—H17B	0.9900
C4—C5	1.418 (2)	C18—H18A	0.9900
C5—C6	1.363 (2)	C18—H18B	0.9900
C5—H5	0.9500	C19—C20	1.521 (2)
C6—C7	1.4145 (18)	C19—H19A	0.9900
C6—H6	0.9500	C19—H19B	0.9900
C7—C8	1.3829 (17)	C20—H20A	0.9900
C7—C10	1.5238 (18)	C20—H20B	0.9900
C8—C9	1.4221 (18)		
			
C8—O1—H1A	113.6 (14)	C12—C11—C10	121.89 (12)
C18—O2—C19	109.25 (12)	C13—C12—C11	120.92 (13)
C1—N1—C9	117.67 (11)	C13—C12—H12	119.5
C20—N2—C17	107.19 (11)	C11—C12—H12	119.5
C20—N2—C10	112.49 (10)	C12—C13—C14	120.22 (13)
C17—N2—C10	109.45 (11)	C12—C13—H13	119.9
N1—C1—C2	124.18 (12)	C14—C13—H13	119.9
N1—C1—H1	117.9	C15—C14—C13	119.30 (8)
C2—C1—H1	117.9	C15—C14—H14	120.4
C3—C2—C1	118.74 (13)	C13—C14—H14	120.4
C3—C2—H2	120.6	C14—C15—C16	120.40 (8)
C1—C2—H2	120.6	C14—C15—H15	119.8
C2—C3—C4	119.70 (13)	C16—C15—H15	119.8
C2—C3—H3	120.1	C15—C16—C11	121.10 (13)
C4—C3—H3	120.1	C15—C16—H16	119.4
C3—C4—C9	117.19 (12)	C11—C16—H16	119.4
C3—C4—C5	124.03 (12)	N2—C17—C18	109.75 (13)
C9—C4—C5	118.78 (12)	N2—C17—H17A	109.7
C6—C5—C4	120.16 (12)	C18—C17—H17A	109.7
C6—C5—H5	119.9	N2—C17—H17B	109.7
C4—C5—H5	119.9	C18—C17—H17B	109.7
C5—C6—C7	122.15 (12)	H17A—C17—H17B	108.2
C5—C6—H6	118.9	O2—C18—C17	111.81 (13)
C7—C6—H6	118.9	O2—C18—H18A	109.3
C8—C7—C6	118.56 (11)	C17—C18—H18A	109.3
C8—C7—C10	119.13 (11)	O2—C18—H18B	109.3
C6—C7—C10	122.28 (11)	C17—C18—H18B	109.3
O1—C8—C7	118.68 (11)	H18A—C18—H18B	107.9
O1—C8—C9	120.63 (11)	O2—C19—C20	112.01 (13)
C7—C8—C9	120.69 (11)	O2—C19—H19A	109.2
N1—C9—C4	122.51 (12)	C20—C19—H19A	109.2
N1—C9—C8	117.86 (11)	O2—C19—H19B	109.2
C4—C9—C8	119.63 (11)	C20—C19—H19B	109.2
N2—C10—C11	112.51 (11)	H19A—C19—H19B	107.9
N2—C10—C7	110.61 (10)	N2—C20—C19	109.38 (12)
C11—C10—C7	109.03 (10)	N2—C20—H20A	109.8
N2—C10—H10	108.2	C19—C20—H20A	109.8
C11—C10—H10	108.2	N2—C20—H20B	109.8
C7—C10—H10	108.2	C19—C20—H20B	109.8
C16—C11—C12	118.06 (13)	H20A—C20—H20B	108.2
C16—C11—C10	119.95 (12)		
			
C9—N1—C1—C2	0.5 (2)	C20—N2—C10—C7	−173.89 (11)
N1—C1—C2—C3	0.4 (3)	C17—N2—C10—C7	67.06 (14)
C1—C2—C3—C4	−0.6 (3)	C8—C7—C10—N2	−155.26 (12)
C2—C3—C4—C9	−0.1 (2)	C6—C7—C10—N2	26.60 (17)
C2—C3—C4—C5	179.03 (16)	C8—C7—C10—C11	80.52 (15)
C3—C4—C5—C6	−179.64 (15)	C6—C7—C10—C11	−97.63 (14)
C9—C4—C5—C6	−0.5 (2)	N2—C10—C11—C16	122.96 (13)
C4—C5—C6—C7	−1.0 (2)	C7—C10—C11—C16	−113.94 (14)
C5—C6—C7—C8	1.5 (2)	N2—C10—C11—C12	−60.73 (15)
C5—C6—C7—C10	179.64 (13)	C7—C10—C11—C12	62.37 (15)
C6—C7—C8—O1	179.42 (12)	C16—C11—C12—C13	0.05 (19)
C10—C7—C8—O1	1.21 (19)	C10—C11—C12—C13	−176.33 (13)
C6—C7—C8—C9	−0.45 (19)	C11—C12—C13—C14	0.4 (2)
C10—C7—C8—C9	−178.66 (12)	C12—C13—C14—C15	−0.71 (17)
C1—N1—C9—C4	−1.3 (2)	C13—C14—C15—C16	0.63 (11)
C1—N1—C9—C8	179.12 (14)	C14—C15—C16—C11	−0.20 (18)
C3—C4—C9—N1	1.1 (2)	C12—C11—C16—C15	−0.1 (2)
C5—C4—C9—N1	−178.08 (14)	C10—C11—C16—C15	176.31 (13)
C3—C4—C9—C8	−179.32 (14)	C20—N2—C17—C18	59.61 (15)
C5—C4—C9—C8	1.5 (2)	C10—N2—C17—C18	−178.12 (11)
O1—C8—C9—N1	−1.29 (19)	C19—O2—C18—C17	57.69 (18)
C7—C8—C9—N1	178.58 (13)	N2—C17—C18—O2	−60.32 (18)
O1—C8—C9—C4	179.12 (12)	C18—O2—C19—C20	−57.41 (16)
C7—C8—C9—C4	−1.0 (2)	C17—N2—C20—C19	−58.99 (15)
C20—N2—C10—C11	−51.69 (15)	C10—N2—C20—C19	−179.34 (12)
C17—N2—C10—C11	−170.73 (11)	O2—C19—C20—N2	59.55 (16)

### Hydrogen-bond geometry (Å, º)

**Table:**

*D*—H···*A*	*D*—H	H···*A*	*D*···*A*	*D*—H···*A*
O1—H1*A*···N1^i^	0.85 (2)	2.01 (2)	2.7668 (14)	148 (18)

Symmetry code: (i) −*x*, −*y*+1, *z*.
